# Supplementary material for: Sharing images of children on social media: British motherhood influencers and the privacy paradox
Source: PLoS One. 2025 Jan 15;20(1):e0314472. doi: 10.1371/journal.pone.0314472 (PMC11734948; doi:10.1371/journal.pone.0314472)
Supplement: S1 Table — (DOCX) [file pone.0314472.s001.docx]

| **Construct** | **Items** | **Response scale** |
| --- | --- | --- |
| Perceived sharenting behaviour [49] | Thinking about the last 2 years of your presence on Instagram, on average, how often have you posted pictures or videos (including Stories) of your child/ren on Instagram? | 5-point: (1) Never – Every day (5). |
| Willingness to share information about child/ren [44] | How willing are you to share the following information about your child/ren on your Instagram profile?  Child's name  Child's age  Child's school name  Photo/video of child's face (full face)  Photo/video of child's partial face  Blurred photo/video of child's face  Photo/video of a tantrum  Photo/video of emotions  Photo/video of a child using toilet  Photo/video of breastfeeding  Photo/video of illness/hospital  Photo/video of child not being clean  Photo/video of child showing naked body parts  Photo/video of a child posing in a silly way | 5-point: (1) Not at all willing to share – Very willing to share (5). |
| Situational Privacy Concerns [49] | Please indicate to what extent you agree or disagree with the following statements about your presence on Instagram:  1. Overall, I see no real threat to the privacy of my children due to my presence on Instagram  2. I know that nothing unpleasant will happen to my children due to my presence on Instagram  3. Overall, I find it safe to publish my child/ren’s personal information on Instagram | 5-point: (1) Strongly disagree- Strongly agree (5) |
